# Punicalagin and Ketogenic Amino Acids Loaded Organic Lipid Carriers Enhance the Bioavailability, Mitochondrial β-Oxidation, and Ketogenesis in Maturing Adipocytes

**DOI:** 10.3390/nano12030368

**Published:** 2022-01-24

**Authors:** Pandurangan Subash-Babu, Nouf Al-Numair, Tahani Almuzaini, Jegan Athinarayanan, Ali Abdullah Alshatwi

**Affiliations:** 1 Department of Food Science and Nutrition, College of Food and Agricultural Sciences, King Saud University, P.O. Box 2460, Riyadh 11451, Saudi Arabia; sbpandurangan@ksu.edu.sa (P.S.-B.); toooota-nm@hotmail.com (T.A.); jathinarayanan@ksu.edu.sa (J.A.); 2 Center for Genomic Medicine, King Faisal Specialist Hospital and Research Centre, Riyadh 11451, Saudi Arabia; alnumair@kfshrc.edu.sa; 3 College of Medicine, Alfaisal University, Riyadh 11451, Saudi Arabia

**Keywords:** punicalagin, ketogenesis, chia seed, NLCs, mitochondrial, fatty acid oxidation

## Abstract

The identification of lipolytic bioactive compounds via the functional stimulation of carbohydrate response element-binding protein-1 (CREBp-1) and AMP-activated protein kinase (AMPK) is most warranted. Nano lipid carriers (NLCs) are currently being considered within drug delivery development as they facilitate controlled drug release and have intracellular bioavailability after encapsulating the active principles with lipid matrix. The present study has been designed to synthesize punicalagin, and ketogenic amino acids (KAA) loaded with organic lipid carriers to optimize the liposome-assisted intracellular delivery’s bioavailability. Punicalagin (PUNI) and KAA (tryptophan, methionine, threonine, lysine, and leucine) were encapsulated with chia seed phospholipids by homogenization, emulsification, and cold ultra-sonication method to obtain nano lipid carriers (NLC). The physicochemical characterization of NLCs has been carried out using Zetasizer, FT-IR, and TEM analysis. Punicalagin and ketogenic amino acid-loaded NLCs (NLC-PUNI-KAA) were identified with an average diameter of 240 to 800 nm. The biosafety of NLC-PUNI-KAA has been evaluated in human mesenchymal stem cells. PI staining confirmed that a 0.4, 0.8 or 1.6μg/dL dose of NLC-PUNI-KAA potentially maintains nuclear integration. NLC-PUNI-KAA treated with maturing adipocytes decreased lipid accumulation and significantly increased the gene expression levels of fatty acid beta-oxidation (PPARγC1α, UCP-1 and PRDM-16) pathways when compared to free PUNI (5 μg/dL) treatment. The lipolytic potential has been confirmed by the functional activation of AMPK and CREBp-1 protein levels. In conclusion, NLC-PUNI-KAA treatment effectively increased mitochondrial efficiency more than free punicalagin or orlistat treated maturing adipocyte. Enhanced lipolysis and decreased hypertrophic adipocyte resulted in decreased adipokine secretion, which has been associated with the suppression of obesity-associated comorbidities and vascular cell inflammation. The bioefficacy and lipolytic potential of water-soluble punicalagin have been improved after functional modification into NLCs.

## 1. Introduction

During fasting, the liver produces 90% of endogenous glucose by gluconeogenesis; the process has been controlled by insulin. If insulin fails to control hepatic glucose production, this eventually ends with fasting hyperglycemia, which develops hepatic insulin resistance and type 2 diabetes (T2D) [1,2]. Hepatic de novo lipogenesis and lipolysis in white adipocytes were increased in insulin resistance conditions. The solid association of hepatic insulin resistance and non-alcoholic fatty liver disease (NAFLD) is exceedingly reproducible as NAFLD is most common in all obese type 2 diabetics, and it is a significant indicator of insulin resistance [3]. This acute effect of insulin alters hepatic glucose metabolism by decreasing glycogen synthesis and glycogenolysis in T2D [4]. This progress decline in hepatic lipid oxidation and ATP turn over, which causes hepatic mitochondrial dysfunction [1].

Diacylglycerol and ceramides are considered lipotoxic lipids, and they have been recognized as a factor contributing to the initiation of insulin resistance in non-adipose tissues [5]. Modulating the levels of the lipotoxic lipids (lipid intermediates) through dietary modifications may control hepatic lipid metabolism, NAFLD, and T2D [6]. Macronutrients have a significant impact on health and well-being. Dietary ketogenic amino acids (KAA) intake decrease lipid synthesis and alter lipid synthesis pathways, which help to overcome hepatic steatosis and insulin resistance [7]. KAA stimulates lipid intermediates into ketone bodies, which have been used as fuel for brain cells during low glucose conditions [8]. Failure or defects in KAA prompts ketone bodies; astrocytes stimulate appetite-promoting hormone ghrelin and arrest lipolysis [9]. The dietary intervention of KAA, such as leucine, produced an improved insulin function, decreased hyperglycemia, and hypercholesterolemia in high-fat diet-induced obesity models [10,11].

Dietary supplementation of combined vitamins, minerals, and phytochemicals improved the intracellular healthy nutrient ratio and decreased biomarkers of atherosclerosis in humans [12]. Numerous in vitro, in vivo and human clinical studies support the preventive effects of dietary natural products against metabolic syndrome and related diseases, including NAFLD and AD [13]. The major bioactive compounds identified with pomegranate (*Punica granatum*) fruit rinds are ellagitannin, ellagic acid, anthocyanin, punic acid, and flavonols. Punicalagin, a unique ellagitannin in pomegranate, is responsible for the antioxidative properties [14]. Punicalagin suppresses obesity, enhances mitochondrial oxidative capacity in obesity-associated non-alcoholic fatty liver disease and obesity-induced inflammatory responses [14,15].

Uptake and bioavailability of phytochemicals in the target cells of the human body facilitate their bio-efficacy on disease control. The bioavailability of phytochemicals was decreased intracellularly due to the intestinal digestion and modification of active sites during endocytosis progress. The nano lipid carriers (NLCs) system comprises of biodegradable and biocompatible lipid materials and surfactants accepted for utilization in various drug delivery systems. The unique advantages of NLCs in the drug delivery system are enhanced loading capacity, prevention of expulsion, more flexibility for modulation of drug release, and the versatile delivery for various routes of administration. In the present study, we aimed to prepare nano lipid carriers (NLCs) with the combination of phytoactive principle punicalagin with ketogenic amino acids to increase the bioavailability of punicalagin to enhance fatty acid beta-oxidation in maturing adipocytes and KAA converts lipid intermediate to ketone bodies.

## 2. Materials and Methods

### 2.1. Cell Culture Materials and Chemicals

Human mesenchymal stem cells (hMSCs) were purchased from ATCC (American Type Culture Collection, Manassas, VA, USA). The cell culture agents, such as fetal bovine serum (FBS) and penicillin-streptomycin were obtained from Hyclone Laboratories, USA. Dulbecco’s modified eagle medium (DMEM), Ethylenediaminetetraacetic acid (EDTA), and trypsin were purchased from Gibco, Paisley, UK. The cell to cDNA synthesis kits and SYBR Green PCR Master Mix were obtained from Qiagen, Hilden, Germany. The assay kits were purchased commercially for β-hydroxybutyrate (ab83390, Abcam, Austria) and acetoacetate (ab180875, Abcam, Austria). The ELISA array-based protein assay kits were purchased from Qiagen (MEH004A, Qiagen, Hilden, Germany). Punicalagin (PUNI), MTT [3-(4,5-dimethylthiazol-2-yl)-2,5-diphenyltetrazolium bromide], Propidium iodide, Oil Red O, Nile red, JC-1 stain, 3-isobutyl-1-methyl-xanthine (IBMX), rosiglitazone, dexamethasone (DEX), human insulin, and all the other chemicals for molecular biology assays were purchased from Sigma-Aldrich (St. Louis, MO, USA). 

### 2.2. Synthesis of Nano Lipid Carrier (NLCs)

Punicalagin (PUNI) and KAA (Tryptophan, Threonine, Methionine, Isoleucine, Leucine, and Lysine) were encapsulated with freshly prepared chia seed phospholipid (containing omega 3 and omega 6) by emulsification method to prepare nanolipid carrier (NLCs) by modified methods of Raju et al. (2021). Briefly, the NLCs preparation was carried out using a hot plate (70 °C) magnetic stirrer for 30 minutes with the fixed rotation. The progress of NLCs fabrication was initiated by dissolving solvent-free chia seed phospholipids (30 mg), 0.25% *w*/*w* of phosphatidylethanolamine, and 0.25 mg of stearic acid into 5 mL of chloroform and methanol (2:1) containing a glass beaker placed on the hot plate (70 °C) with stirring. Then, PUNI (0.1, 0.2 and 0.4 μg, respectively) and KAA (0.25 mg) were added drop by drop into the lipid phase. Immediately, the warm organic lipid phase was emulsified with an aqueous phase containing 15 mL of Tween 80 (30 mg). The prepared oil in water dispersion was sonicated in an ice-cold condition, with the frequency of 0.5 cycles and 60% amplitude (using a probe-type ultrasonicator, Sonics, New town, NY, USA). 30 mL volume of NLCs consists of 0.08% PUNI and KAA, 15 mL of an aqueous phase, and 30 mg of lipid phase. The formulation was transferred into the brown glass container and stored in the freezer at 2–4 °C. In separate, PUNI-free NLC-formulation has been prepared to determine the comparative effect.

### 2.3. Physiochemical Characterization of NLCs-PUNI-KAA and NLCs-KAA

Spectrophotometrically, total drug content was estimated at 420 nm by disrupting 1 mL of the NLCs-PUNI-KAA and NLCs-KAA dispersion on the same day of preparation. The chemical interaction between lipid and aqueous phase was identified by FT-IR (Agilent, Santa Clara, CA, USA). Prepared NLCs particle size as z-average diameter was determined using Zetasizer (NANO-Zs90). NLCs samples were loaded (0.5 μL) on the surface of a 300-mesh carbon coated copper grid, and negative staining [2% uranyl acetate (*w*/*v*)] has been used to determine the shape, size, and morphology in high-resolution transmission electron microscopy (HR-TEM, JEOL, Tokyo, Japan).

### 2.4. Human Mesenchymal Stem Cells (hMSCs) Culture and Induction of Adipocyte Differentiation

Cells (hMSCs) were cultured in the growth medium containing DMEM with 10% fetal bovine serum and 100 U/mL penicillin-streptomycin in a humidified 5% CO_2_ incubator at 37 °C. In 96-well or 24 well plates, hMSCs were seeded (1 × 10^4^ cells/well) with standard growth conditions and allowed to reach 80% confluence. After confirming 70% visual confluence (day 0), the media was replaced with adipocyte differentiation media (containing DMEM with 10% FBS, 0.5 mM IBMX, 1 µM dexamethasone 10 μg/mL insulin) and allowed to grow for 3 days. On the 4th day, the media was replaced with an adipogenesis maintenance medium (containing 10% DMEM with 10 μg/mL insulin) and maintained for 2 days (Subash-Babu et al., 2020). Preadipocytes cultured with a maintenance medium were used as a control for all assays.

### 2.5. Cytotoxicity Analysis in hMSCs and Preadipocytes

hMSCs and differentiated preadipocytes were cultured in 96 well culture plate (5000 cells/well), then allowed to reach 80% confluence with respective medium. Further, culture medium containing punicalagin (PUNI), NLCs-PUNI-KAA and NLCs-KAA (0.1, 0.2, 0.4, 0.8, 1.6, 3.2 and 6.4 µg/dL) were treated to respective well and untreated cells were maintained as control, the cells were incubated for 24 h and 48 h, respectively. After incubation, 20 µL of 5 mg/mL MTT (3-[4, 5-dimethylthiazol-2-yl]-2, 5-diphenyltetrazolium bromide) have been added to each well and incubated for 4 h in the dark at 37 °C in a CO_2_ incubator. The development of visible purple formazan crystals was dissolved with 100 µL of DMSO (100%). Absorbance was measured at 570 nm using a microplate reader (Thermo Scientific, Waltham, MA, USA). Percentage of cell viability was calculated by the mean values of absorbance of the test sample/ absorbance of the untreated control) × 100.

### 2.6. Experimental Design for the Inhibition of Lipid Accumulation in Preadipocytes

To determine the bioefficacy, hMSCs were treated with NLCs-PUNI-KAA (0.8 and 1.6 µg/dL), NLCs-KAA (0.8 and 1.6 µg/dL), PUNI (5 μg/dL) and orlistat (6 µM) for 48 h. The control and experimental cells were processed for cell and nuclear morphology, immunomodulatory, and antioxidant gene expression analysis.

To explore the lipolytic potential and determine the regulatory effect on fatty acid metabolism, the preadipocytes were used. As such, on the day-0, adipocyte differentiation was induced to hMSCs and vehicle control was maintained according to the adipocyte differentiation protocol (Section 2.4). On day 4, NLCs-PUNI-KAA (0.8 and 1.6 µg/dL), NLCs-KAA (0.8 and 1.6 µg/dL), PUNI (5 μg/dL) and orlistat (6 µM) were treated with preadipocytes. On day 6, experimental cells were replaced with maintenance medium containing PUNI, NLCs-PUNI-KAA, NLCs-KAA, orlistat and allowed to grow until day 9. On day 10 and 12, the media in each well were replaced with a maintenance medium. The vehicle control was replaced with a maintenance medium without drugs all the time.

On day 14 (end of the experiment), the experimental cell’s condition media (containing PUNI, NLCs-PUNI-KAA, NLCs-KAA, and orlistat treated adipocyte secreted and cellular proteins) have been collected for protein quantification. The adherent cells were used for lipid accumulation analysis and cDNA synthesis for gene expression analysis.

### 2.7. Cell Membrane and Nuclear Damage Analysis in hMSCs

Vehicle control, PUNI, NLCs-PUNI-KAA and NLCs-KAA treated hMSCs were utilized to analyze the apoptotic and necrotic characteristic morphology using inverted fluorescent microscopy after propidium iodide (PI) staining, as described by Leite et al. [16].

### 2.8. Oil Red O and Nile Red Staining Analysis to Determine Intracellular Lipid Levels in Maturing Adipocytes

Intracellular lipid accumulation and lipid droplets were determined by Oil Red O and Nile Red staining according to the previously established method by Subash-Babu et al. [17]. Briefly, the stock solution was prepared by dissolving 250 mg of Oil Red O into 50 mL of 100% isopropanol, and the working solution was prepared with stock and 60% isopropanol with a 3:2 ratio. Vehicle control, PUNI, NLCs-PUNI-KAA, NLCs-KAA, and orlistat treated adipocytes were fixed with paraformaldehyde (4%), then overlaid with working Oil Red O solution (200 μL) for incubation at room temperature for 1 h. After incubation, the free Oil Red O stains were removed by PBS washing; stained cells were analyzed using an inverted light microscope, and images were captured immediately.

For fluorescent Nile Red staining, vehicle control, PUNI, NLCs-PUNI-KAA, NLCs-KAA, and orlistat treated experimental cells were fixed with 4% formaldehyde and stained with Nile Red (5 mg in 1 mL of 100% acetone) for 30 minutes at room temperature. After incubation, the fluorescence lipid droplets were immediately captured under an inverted fluorescence microscope.

### 2.9. Mitochondrial Membrane Potential (Δψ_m_) (JC-1 Staining) Assay

Mitochondrial membrane potential (Δψ_m_) was determined in vehicle control, PUNI, NLCs-PUNI-KAA, NLCs-KAA, and orlistat treated adipocytes to assess mitochondrial functional capacity using JC-1 assay. Briefly, the experimental cells were overlaid with JC-1 staining solution and incubated for 20 min in the dark at room temperature. After incubation, 200 μL of JC-1 stain wash buffer was used to remove the unbound JC-1 dye. Then, the appearance of red and green fluorescence was observed using a fluorescence microscope, and images were captured.

### 2.10. Estimation of Triglyceride (TG), Total Cholesterol and Free Glycerol

The amount of triglyceride (TG), total cholesterol, and free glycerol in vehicle control, NLCs-PUNI-KAA, NLCs-KAA, PUNI, and orlistat treated maturing adipocytes were measured using the commercial kit method (Abcam, Austria) [18]. The protein content in adipocyte cells was determined according to the Bradford method [19].

### 2.11. Quantitative Polymerase Chain Reaction (qPCR) Analysis

The cDNA has been synthesized from experimental cells after isolation of total RNA using Fastlane^®^ Cell cDNA isolation kit using qPCR instrument setup. The levels of oxidative stress [LPO (lipid peroxidation), NOS (Nitric oxide synthase)], antioxidant [CYP1A (Cytochrome *P450* family *1* subfamily A member *1*), GSK-3β (Glycogen synthase kinase 3 Beta), GPx-1 (glutathione peroxidase-1)] and tissue damage [TNFα (tissue necrosis factor-alpha), NF-κb (nuclear factor kappa B), IL-1β (interleukin-1 beta), Inf-γ (Interferon-gamma)] associated mRNA expression levels in hMSCs; maturing adipocyte’s fatty acid oxidation [Adiponectin-R1, PPARγC1α (peroxisome proliferator activated receptor gamma coactivator 1 alpha), UCP-1 (uncoupling protein-1), PRDM16 (PR domain containing protein 16)], insulin resistance and metabolic inflammation (IL1β, IL-4 (Interleukin-4), TNFα, NF-κB) related genes expressions and the reference gene, β-actin have been analyzed by the method of Yuan et al. [20]. The amplification values (ΔCt) have been calculated by the variance between Ct (treated) and Ct (control). Gene expressions were plotted using the expression of 2^−ΔΔCt^ value.

### 2.12. Quantification of CREBp-1 and AMPK Protein by ELISA

The amount of fatty acid metabolism associated signaling proteins such as CREBp-1 (Carbohydrate response element binding protein-1) and AMPK (AMP-activated protein kinase) in adipocytes were analyzed in vehicle control, PUNI, NLCs-PUNI-KAA, NLCs-KAA and orlistat treated cells using high-sensitivity ELISA-kits (Quantikine, R&D Systems, Minneapolis, MN, USA). This assay does not distinguish between soluble and receptor-bound proteins, and it gives a measure of the total concentration of proteins. The values were expressed as pg/mg protein for all the analyzed proteins.

### 2.13. Estimation of Acetoacetate (AcAc) and Beta-Hydroxybutyrate (β-HB)

Colorimetric acetoacetate (AcAc) assay kit (ab180875, Abcam, Austria) has been used to quantify the endogenous levels of AcAc in condition media of vehicle control, PUNI, NLCs-PUNI-KAA, NLCs-KAA, and orlistat treated adipocytes. In this non-enzymatic assay, an unknown sample containing AcAc was reacted with a substrate to generate a colored product after incubation for 10 to 15 min at 25 °C (protect the plate from light). The absorbance can be measured at 550 nm. The assay kit can detect samples containing acetoacetate as low as 25 µM. The reaction is specific for AcAc and does not interact with 3-β-hydroxybutyrate content.

The quantity of β-hydroxybutyrate (β-HB) in condition media was measured with an analyzer (Cobas-bio-centrifugal analyzer, Roche Diagnostics, Somerville, NJ, USA) with commercially established liquid reagent (ab83390, Abcam, Austria) containing β-HB dehydrogenase and nicotinamide-adenine dinucleotide as described by Williamson et al. [21]. During the assay, the β-HB dehydrogenase removes hydrogen molecule from nicotinamide-adenine dinucleotide and supply to β-HB. The amount of reduced nicotinamide-adenine dinucleotide was monitored by measuring the 340-nm absorption, which was directly proportional to the β-HB concentration in the sample.

### 2.14. Statistical Analysis

The statistical evaluation was carried out using the SPSS/28.5 software package. The values were analyzed by one-way analysis of variance (ANOVA) followed by Tukey’s range test. All the experimental grouped data and results were expressed as mean ± SD for six replications in each group. The *p* values ≤ 0.05 and ≤0.001 were considered significant [22].

## 3. Results

### 3.1. Characterization of NLCs-PUNI-KAA and NLCs-KAA Using Zetasizer, FT-IR, and TEM Image Analysis

**Figure 1:**
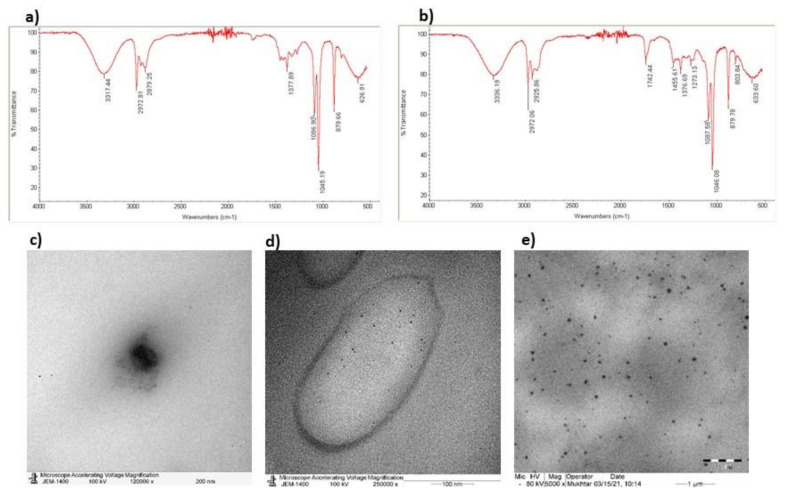
FT-IR spectra of ketogenic amino acid loaded chia seed phospholipids [NLCs-KAA] (**a**), punicalagin, ketogenic amino acids loaded chia seed phospholipids [NLCs-PUNI-KAA] (**b**) containing nano lipid carriers. TEM images of chia seed phospholipid matrix (**c**), NLCs-KAA (**d**), NLCs-PUNI-KAA (**e**) containing nano lipid carriers.

Active principle punicalagin encapsulation efficiency has been confirmed by identifying functional groups using FT-IR data (Figure 1a,b). TEM analysis confirmed the chia seed oil dispersion size (Figure 1c), and individual particle morphology remained the same in the range of 300 to 800 nm (Figure 1d,e). The comparison FT-IR data between PUNI, NLCs-PUNI-KAA, and NLCs-KAA confirmed no significant loss or missing peak. The appearance of a new peak such as 3336.1 cm^−1^ (N-H stretching, aliphatic amine group), 2925.8 cm^−1^ (C-H stretching in the aliphatic group), 1742.4 cm^−1^ (C=O stretching aldehyde or amine group) and 1455.6 cm^−1^ (C-H bending in alkane of methyl group) in NLCs-PUNI-KAA, confirmed the addition of PUNI functional groups. The average particle size was found in Zetasizer as 300 to 800 nm for NLCs-KAA, however an encapsulation of NLCs-PUNI-KAA encapsulated NLCs was found with 450 to 850 nm (Supplementary Figure S1b,c). Furthermore, the higher entrapment of PUNI controlled the oil in the drug release from the encapsulated nano lipid carriers.

### 3.2. Effect of Punicalagin (PUNI), NLCs-PUNI-KAA, and NLCs-KAA on Cell Proliferation Potential in hMSCs and Adipocytes

**Figure 2:**
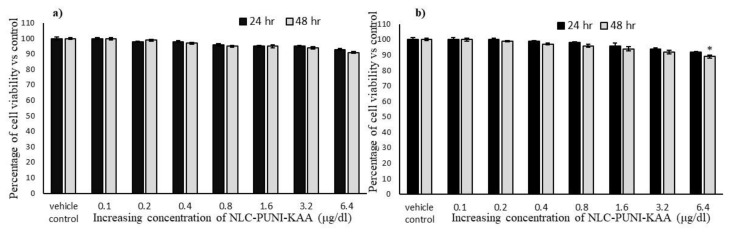
In vitro cytotoxic effect of vehicle control and NLC-PUNI-KAA on human mesenchymal stem cells (**a**) and adipocytes (**b**) after 24 h and 48 h. Each values are means ± SD (*n* = 6). * representing a significance level of *p* ≤ 0.05 by comparison with vehicle control.

To determine the effect of a testing drug on cell proliferation or growth inhibition potential, we treated with increasing concentration (0.1, 0.2, 0.4, 0.8, 1.6, 3.2 and 6.4 µg/dL) of punicalagin (PUNI), NLCs-PUNI-KAA and NLCs-KAA to hMSCs or adipocyte’s and allowed to grow for 24 h or 48 h, respectively. After 48 h incubation, a tested higher dose (6.4 µg/dL) of NLCs-PUNI-KAA showed a minimal percentage of growth inhibition or cell death both in hMSCs (9%) and preadipocytes (11%) (Figure 2a). Even after 48 h, no significance on viability inhibition was observed in NLCs-PUNI-KAA treated hMSCs and preadipocytes (Figure 2b). However, PUNI and NLCs-KAA treatment produced 6% and 13% in hMSCs; 14% and 21% of cell viability inhibition was observed in preadipocytes after 48 h, respectively (Supplementary Figure S3).

### 3.3. Biosafety Analysis of NLCs-PUNI-KAA, NLCs-KAA, and PUNI in hMSCs Using Propidium Iodide (PI) Staining

Propidium iodide (PI) staining has been used to determine the effect of NLCs-PUNI-KAA and NLCs-KAA on nuclear damage morphology when compared with PUNI alone-treated hMSCs. Vehicle control and NLCs-treated experimental cells showed regular morphology without any uneven shape or damaged nucleus. The results were compared with the PUNI drug alone-treated hMSCs (Supplementary Figure S3).

### 3.4. Effective Dose Determination Based on Lipid Accumulation Inhibitory Potential

**Figure 3:**
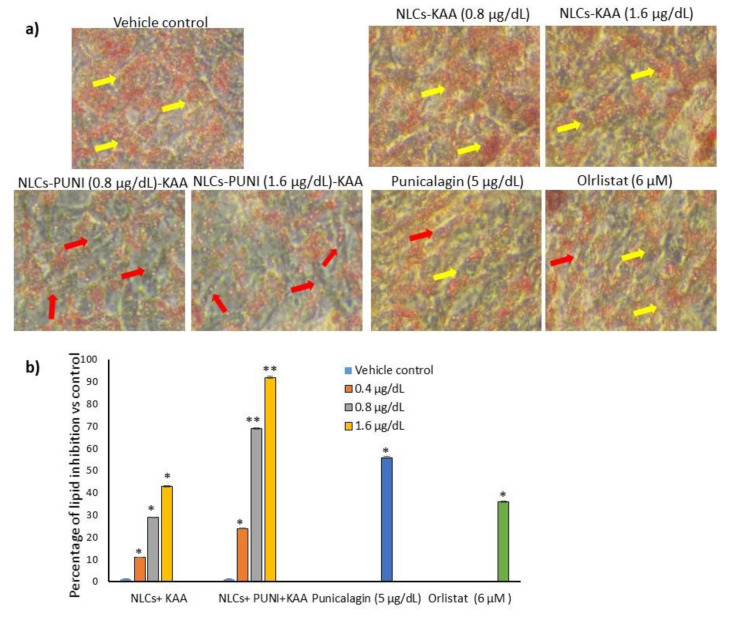
Oil Red O stained light microscopic image (**a**) of vehicle control, NLCs-KAA, NLCs-PUNI-KAA, PUNI and orlistat treated maturing adipocyte after 14 days. The quantity of Oil Red O stain in each group (**b**) directly resembles the lipid accumulation level. Each of the values are means ± SD (*n* = 6). ** *p* ≤ 0.001 and * *p* ≤ 0.05 compared with vehicle control. In Oil Red O staining, vehicle control showing hypertrophic adipocyte (yellow arrow heads) directly propositional to excessive triglyceride storage. However, in NLCs-PUNI-KAA treatment found with less lipid accumulation and linear adipocytes morphology (red arrow heads). NLCs-PUNI-KAA treated cells showing highest inhibition of lipid accumulation compared to NLCs-KAA or PUNI or orlistat.

In the present study, 0.2, 0.4, 0.8, and 1.6 μg/dL concentrations of punicalagin (PUNI), NLCs-PUNI-KAA, and NLCs-KAA were selected and treated to maturing adipocytes to assess lipid accumulation inhibition potential as per the experimental protocol. After 14 days of treatment, Oil Red O staining (Figure 3a) images confirmed that 0.8 and 1.6 μg/dL doses of NLCs-PUNI-KAA were found with linear-shaped adipocytes (red arrowheads) and reduced lipid accumulation when compared to PUNI and NLCs-KAA (identified with hypertrophic adipocytes-yellow arrowheads). Even though lipid accumulation was found to be notable in PUNI (5 μg/dL) and NLCs-KAA (0.8 and 1.6 μg/dL doses) when compared with untreated control (hypertrophic adipocytes indicated with yellow arrowheads). Observed results have found that the lipid content was significantly (*p* ≤ 0.001) decreased by 69% in 0.8 μg/dL and 92% in 1.6 μg/dL in NLCs-PUNI-KAA when compared with the vehicle control (Figure 3b). Comparatively, NLCs-KAA and PUNI inhibition levels were found as 43% vs. 56%. The reference drug orlistat (6 μM) decreased 36% lipid content only after 14 days compared to vehicle control.

### 3.5. Identification of Hypertrophic Adipocyte Using Nile Red Fluorescence Staining Analysis

**Figure 4:**
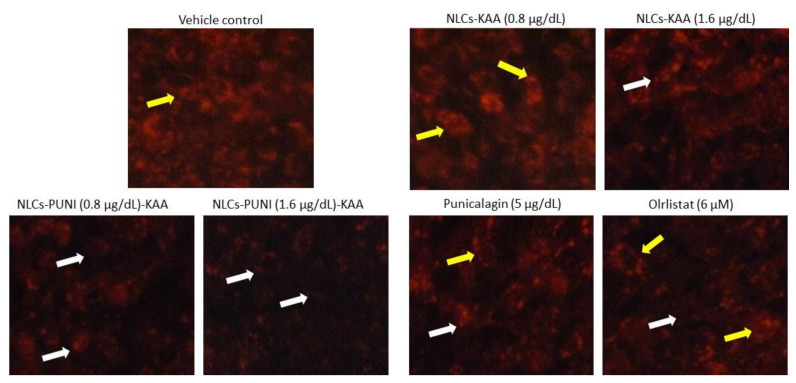
Nil Red fluorescence stained images of vehicle control, NLCs-KAA, NLCs-PUNI-KAA, PUNI and orlistat treated maturing adipocyte after 14 days. Images showing less lipid accumulation in NLCs-PUNI-KAA when compared to all other treatments. In Nile Red fluorescence staining, vehicle control showing high red fluorescence directly proportional to intracellular lipid accumulation level (yellow arrow head). However, in NLCs-PUNI-KAA treatment found with less red fluorescence confirmed low levels of intracellular lipid accumulation (white arrow head). 1.6 µg/dL dose of NLCs-PUNI-KAA treatment showing highest inhibition of lipid accumulation compared to NLCs-KAA or PUNI or orlistat.

The Nile Red staining confirms the hypertrophic and high lipid accumulation in untreated adipocytes after 14 days. As such, 0.8 and 1.6 μg/dL of NLCs-PUNI-KAA, NLCs-KAA and PUNI treated maturing adipocytes shown significantly (*p* ≤ 0.001) decreased the red fluorescence, which represents the lowest lipid content and hypertrophy morphology (Figure 4). The lipid content inhibitory effect of NLCs-PUNI-KAA was found to be more significant than NLCs-KAA. Most notably, 5 mg/dL PUNI or 6 µM of orlistat did not produce significant inhibition of lipid content or adipocyte hypertrophy; also the effect was lower than the 0.8 µg/dL dose of NLC-PUNI-KAA (*p* ≤ 0.05).

### 3.6. Mitochondrial Membrane Potential (Δψ_m_, JC-1) and Oxidative Capacity Analysis

**Figure 5:**
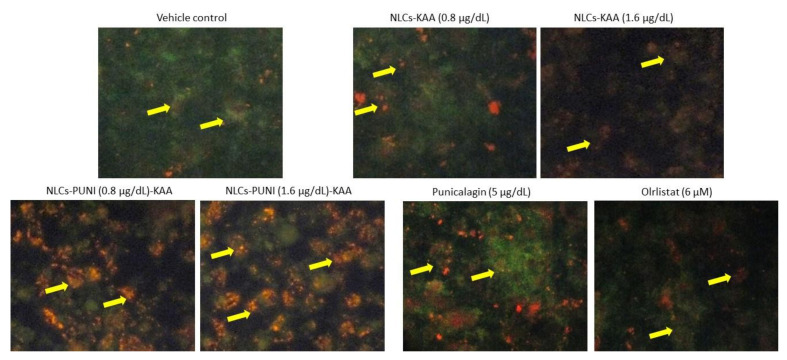
Determination of mitochondrial membrane potential (MMP, Δψ_m_) in vehicle control, NLCs-KAA, NLCs-PUNI-KAA, PUNI and orlistat treated maturing adipocyte after 14 days. Fluorescence images showing merged features of the red and green signals of the JC-1 dye, corresponding to J-aggregates vs. JC-1 monomeric form. In vehicle control, maturing adipocytes found with JC- monomers (green fluorescence), confirmed depolarized mitochondrial membrane potential (Δψ_m_) with low mitochondrial efficiency. We found less J-aggregates and depolarized mitochondria in vehicle control, NLCs-KAA and orlistat treated groups. In NLCs-PUNI-KAA (1.6 μg/dL) treated adipocyte showing high j-aggregates (red fluorescence) directly representing active mitochondria (high MMP, Δψ_m_) than 0.8 μg/dL or NLC-KAA or free punicalagin treatment.

Mitochondrial membrane potential (Δψ_m_) is the predicted mitochondrial oxidative capacity for fatty acid energy metabolism. Figure 5 shows the images of JC-1 staining for vehicle control, NLCs-PUNI-KAA, NLCs-KAA, PUNI and orlistat treated maturing adipocytes, clearly representing merged images of JC-1 dye having red and green signals, corresponding to JC-1 monomeric form vs. J-aggregate. We found that a 1.6 µg/dL dose of NLCs-PUNI-KAA and NLCs-KAA resulted in high J aggregates (red fluorescence) that directly represent the polarized mitochondrial that correspond to potential mitochondrial oxidative capacity and thermogenesis. The vehicle control or PUNI or orlistat treated maturing adipocytes found with JC-monomers (green fluorescence) confirmed a depolarized mitochondrial membrane potential (Δψ_m_) with low mitochondrial efficiency.

### 3.7. Intracellular Total Cholesterol, TG, and HDL Levels

**Figure 6:**
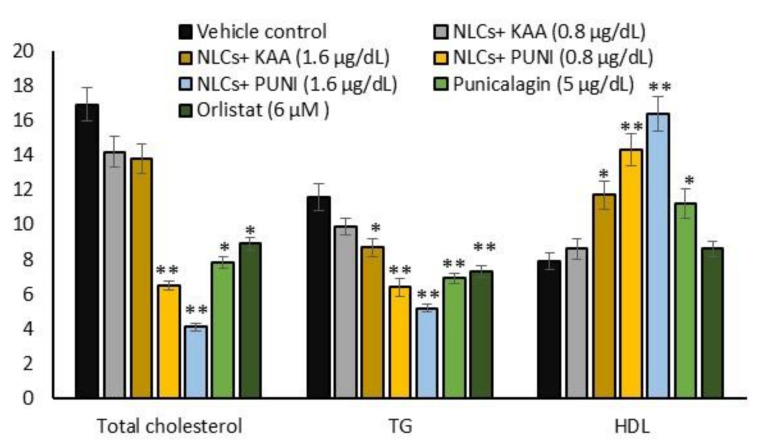
Changes in total cholesterol, triglycerides (TG) and HDL levels in vehicle control, NLCs-KAA, NLCs-PUNI-KAA, PUNI and orlistat treated maturing adipocyte after 14 days. Each of the values are means ± SD (*n* = 6). Significance levels are represented as ** *p* ≤ 0.001 and * *p* ≤ 0.05 compared with vehicle control.

The lipid-lowering effect has been confirmed by an intracellular accumulated amount of total cholesterol, TG, and free HDL levels in NLCs-PUNI-KAA and NLCs-KAA treated maturing adipocytes were shown in Figure 6. Treatment with 1.6 µg/dL of NLCs-PUNI-KAA and NLCs-KAA significantly reduced the total cholesterol, TG, and increased HDL levels in adipocytes, when compared with PUNI or orlistat, treated maturing adipocytes after 14 days.

### 3.8. Oxidative Stress, Antioxidant and Tissue Damage Related Gene Expressions in NLCs-PUNI-KAA, NLCs-KAA, and PUNI Treated hMSCs

**Figure 7:**
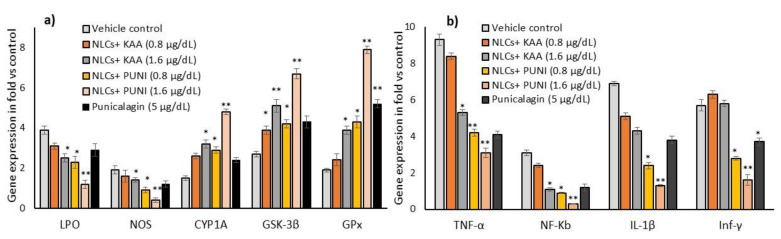
Alterations in oxidative stress (**a**) and tissue damage (**b**) associated mRNA expression levels in vehicle control, NLCs-KAA, NLCs-PUNI-KAA and PUNI treated hMSCs after 2 days. Each of the values are means ± SD (*n* = 6). Significance levels are represented as ** *p* ≤ 0.001 and * *p* ≤ 0.05 compared with vehicle control (normalized with β-actin).

In hMSCs, alterations in oxidative stress and proinflammatory cytokine-related mRNA expressions were analyzed after 48 h of NLCs-KAA, NLCs-PUNI-KAA, PUNI treatment. The observed results confirmed that NLCs-PUNI-KAA treated hMSCs significantly decreased the LPO, NOS and increased the antioxidant genes such as GSK-3β, CYP1A, GPx expressions (Figure 7a). In addition, the oxidative stress-induced cell and tissue necrosis associated proinflammation related genes such as TNF-α, NF-κB, IL-1β, and Inf-γ expressions were decreased in NLCs-PUNI-KAA treated hMSCs (Figure 7b). The observed effect of NLCs-PUNI-KAA was more significant than NLCs-KAA and Punicalagin treated hMSCs.

### 3.9. Adipogenesis, Mitochondrial Thermogenesis and Inflammatory Gene Expression Analysis in NLCs-PUNI-KAA, NLCs-KAA, PUNI, and Orlistat Treated Maturing Adipocytes

**Figure 8:**
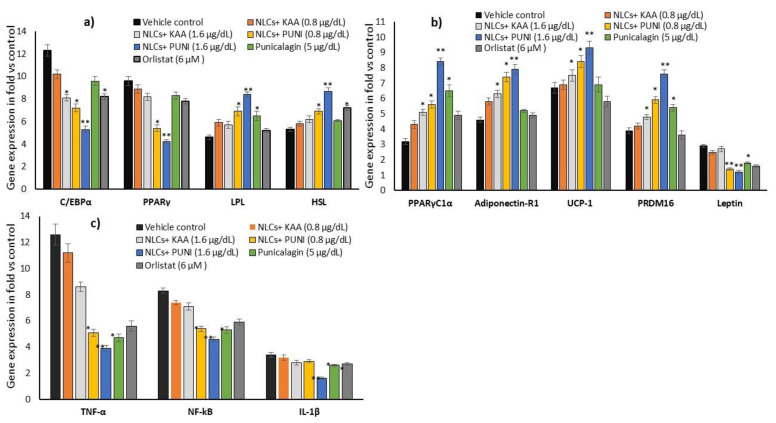
Changes in the adipogenesis (**a**), mitochondrial fatty acid oxidative (**b**) and adipokine (**c**) associated mRNA expression levels in vehicle control, NLCs-KAA, NLCs-PUNI-KAA and PUNI and orlistat treated maturing adipocyte after 14 days. Each of the values are means ± SD (*n* = 6). Significance levels are represented as ** *p* ≤ 0.001 and * *p* ≤ 0.05 compared with vehicle control (normalized with β-actin).

Gene expression levels in NLCs-PUNI-KAA, NLCs-KAA, PUNI and orlistat treated maturing adipocyte after 14 days showed significantly (*p* ≤ 0.001) decreased adipocyte differentiation-related C/EBPα, PPARγ and increased lipoprotein lipase, hormone-sensitive lipase mRNA levels in NLCs-PUNI-KAA (1.6 µg/dL), when compared to NLCs-KAA, PUNI or orlistat treated maturing adipocytes (Figure 8a). Figure 8b shows the adipocyte’s mitochondrial efficiency-related mRNAs such as PPARγC1α, Adiponectin-R1, UCP-1, PRDM-16 have been significantly increased, and leptin expression was found to be decreased in NLCs-PUNI-KAA treated maturing adipocytes. The metabolic inflammation-related genes such as TNF-α, NF-κB, and IL-1β expressions have been decreased in NLCs-PUNI-KAA (1.6 µg/dL), when compared to NLCs-KAA, PUNI or orlistat treated maturing adipocytes (Figure 8c). All the gene expression levels were significantly increased in a higher dose of 1.6 µg/dL compared to the lower dose of 0.8 µg/mL of NLCs-PUNI-KAA.

### 3.10. Intracellular Protein Levels in Adipocyte’s Stromal Vascular Fraction (SVF)

**Figure 9:**
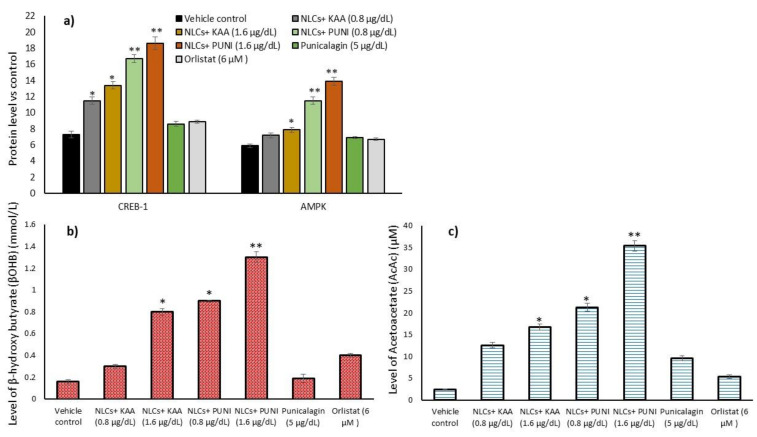
Alterations in CREBp-1, AMPK protein (**a**), metabolic intermediates beta-hydroxybutyrate (**b**) and acetoacetate (**c**) levels in vehicle control, NLCs-KAA, NLCs-PUNI-KAA and PUNI and orlistat treated maturing adipocyte after 14 days. Each of the values are means ± SD (*n* = 6). Significance levels are represented as ** *p* ≤ 0.001 and * *p* ≤ 0.05 compared with vehicle control.

The results of adipocyte’s mitochondrial thermogenesis (CREBp-1 and AMPK) related protein expression levels of NLCs-PUNI-KAA, NLCs-KAA, PUNI (1.6 µg/dL) and orlistat (6 µM) treated adipocyte microparticles are shown in Figure 9a. We found significantly (*p* ≤ 0.001) increased levels of CREBp-1 and AMPK and decreased NF-kB and TNF-α levels of NLCs-PUNI-KAA treatment, compared to NLCs-KAA, PUNI, or orlistat treatment after 14 days. The observed finding confirmed the gene expression patterns of increased mitochondrial oxidative capacity and decreased inflammatory conditions in mature adipocytes.

### 3.11. Acetoacetate and Beta-Hydroxybutyrate Levels

Maturing adipocytes prone to fatty acid oxidation stimulation, intermediate metabolites such as acetoacetate and β-hydroxybutyrate (ketone bodies) have been produced. In the present study, we analyzed the acetoacetate and β-hydroxybutyrate levels in maturing adipocytes after NLCs-PUNI-KAA treatment, and the results were compared with vehicle control, NLCs-KAA, PUNI, and orlistat treated maturing adipocytes. Beta-hydroxybutyrate (Figure 9b) and acetoacetate (Figure 9c) were significantly increased in the 1.6 µg/dL dose NLCs-PUNI-KAA treated adipocytes when compared with 0.8 µg/dL of NLCs-PUNI-KAA or 1.6 µg/dL of NLC-KAA or 5 mg/dL PUNI or 6 µM of orlistat treated groups. The observed result confirmed the 1.6 µg/dL of NLCs-PUNI-KAA induced fatty acid oxidation in maturing adipocytes was evidenced by the fatty acid oxidation associated with converted ketone body metabolites.

## 4. Discussion

Pharmacological agents that stimulate mitochondrial fatty acid oxidation and utilize the lipid intermediates to synthesize ketone bodies in fasting might be more beneficial for controlling hepatic glucose production, insulin resistance, and type 2 diabetes risk. KAA aid the clearance of lipotoxic lipids via the stimulation of the ketogenesis pathway. Solerte et al. [23] have found that oral administration of leucine, isoleucine, valine, threonine, and lysine containing KAA mixture improved insulin sensitivity in elderly patients with type-2 diabetes. In particle size and TEM analysis, the fabricated nano lipid carriers (NLCs) with punicalagin and ketogenic amino acids have been identified with uniform shape with 200 to 800 nm range. FT-IR confirmed the encapsulation of punicalagin internally to the chia seed phospholipid-based nano lipid carrier. NLC has the higher loading capacity of the drug (dissolved in oil) and is encapsulated with solid lipid, which aids the controlled drug release [24]. In addition, NLC improves chemical stability, high bioavailability, and the slow release of functional lipophilic compounds from food, easily entrapped intracellularly without conformational changes of extracellular enzymatic digestion [25]. The biosafety analysis of NLCs-PUNI-KAA did not inhibit the cell proliferation or induce cytotoxicity in human mesenchymal stem cells, and preadipocytes with the tested higher concentration. In this context, McDonald and Cervenka [26] have evidenced the ketogenic diet feasibility, safety, and potential efficacy in the management of status epilepticus.

NLCs-PUNI-KAA treatment decreased lipid accumulation was confirmed by reduced lipid droplets in Nile Red staining after 14 days in matured adipocytes. NLCs-PUNI-KAA treated maturing adipocytes confirmed enhanced mitochondrial membrane potential and healthy mitochondria in the JC-1 assay. NLC-KAA treated cells identified with lipid droplets and lesser mitochondrial membrane potential ensure the decreased mitochondrial oxidative capacity. Punicalagin combined with KAA attenuate mitochondrial oxidative capacity and energy production via lipolysis; this might be due to the punicalagin mediated enhanced fatty acid oxidation and energy production capacity. In this context, Binyamin et al. [27] have confirmed that pomegranate seed oil containing nanodroplets delays mitochondrial damage and prevents neurodegenerative disease onset. In addition, pomegranate vinegar decreased adiposity by stimulating fatty acid oxidation in the liver [28]. Urolithin A, a phytoactive principle from pomegranate, increases energy expenditure by enhancing thermogenesis in brown adipose tissue and inducing the browning of white adipose tissue [29].

The mitochondrial energy production capacity of NLCs-PUNI-KAA was further confirmed by increased gene expression levels associated with lipolysis and thermogenesis. Adipocyte thermogenesis stimulating mRNAs such as PPARγC1α, UCP-1, and PRDM-16 have been elevated, and lipogenesis-associated SREBP-1 expression was decreased. Notable, adipogenesis was suppressed; this was confirmed by the decreased C/EBP-α, PPAR-γ and increased LPL and HSL mRNA expression levels. NLCs-PUNI-KAA treatment increased the protein expression levels of adipocyte metabolism associated with AMP-activated protein kinase (AMPK) and CREBp-1 to twofold. In the present study, enhanced AMPK phosphorylation arrested adipocyte maturation by downregulating the central regulators of adipogenesis (C/EBPα and PPARγ) in mature adipocytes. Notably, pomegranate leaf extract (PLE) is confirmed to be a novel appetite suppressant that only affects obesity due to a high-fat diet [30,31].

The upregulated CREBp-1 activity via the cAMP–PKA pathway has been confirmed by the stimulated PPARγC_1_α, which further activated the thermogenic genes PRDM16 and UCP_1_ [32]. In fasting conditions, cellular ATP depletion activates AMPK, which increases AMP/ATP ratio and initiates metabolic and genetic events to restore ATP levels via fatty acid beta-oxidation [33]. A phosphorylated form of AMP-activated protein kinase was identified upon stimulated lipolysis in adipocytes, which decreases the lipid content via thermogenesis [34]. AMPK upregulates ketogenesis in glial cells via the promotion of fatty acid breakdown, which aids ketone bodies synthesis in astrocytes [7]. AMPK dependent ketone bodies synthesis has been mediated by the subsequent inhibition of a fatty acid synthesizing enzyme-acetyl CoA carboxylase (ACC), which prevents the synthesis of malonyl CoA, an inhibitor of carnitine-palmityl transferase-I [35]. Treatment with NLCs-PUNI-KAA to adipocytes indirectly modulates the AMPK/ACC axis and contributes to the activation of CPT-1, which imports free fatty acids to mitochondria and aids the progression of mitochondrial fatty acid oxidation. In this context, Hasegawa et al. [36] have confirmed that the ketone body utilizing enzyme acetoacetyl-CoA synthetase, functionally attenuated in 3T3-L1 adipocyte. The metabolism of ketone bodies and their fuel mechanism in adipocytes and hepatocytes have been well explored [37]. 

In contrast, in the present study, NLC-KAA treated adipocytes, the expression levels of thermogenesis, and suppressed adipogenesis-related mRNA have not been well attenuated. This is despite previous findings having identified that feeding with leucine alone controlled a high-fat diet induced obesity, hyperglycemia, and hypercholesterolemia in mice [10,11]. The combination of punicalagin with KAA (tryptophan, threonine, leucine, and lysine) effectively attenuated mitochondrial membrane potential and increased adipocyte thermogenesis-associated gene expressions. In this context, Yeh [38] has confirmed that leucine’s catabolism in adipocytes was converted into ketone bodies such as acetoacetate and β-hydroxybutyrate. KAA stimulates lipid intermediates into ketone bodies used as fuel for brain cells during low glucose conditions [36]. The present study found increased acetoacetate levels and β-hydroxybutyrate levels in NLCs-PUNI-KAA treated cell condition media. Also, intracellular triglyceride and total cholesterol levels were decreased in NLCs-PUNI-KAA treated maturing adipocytes when compared to vehicle control or NLCs-KAA treatment. Failure or defects in KAA stimulated ketone bodies, or astrocytes, facilitate the appetite-promoting hormone ghrelin and arrest lipolysis [9]. In addition, the removal of lysine or threonine from a diet increases hepatic lipid storage and NAFLD in rodents [39].

## 5. Conclusions

Obesity is established as the metabolic syndromes that develop due to a nutritional imbalance or inadequate diet. The edible fruit pomegranate, having the phytoactive principle punicalagin, is a well-known antioxidant and mitochondrial oxidative capacity increasing compound. This punicalagin combined with KAA (tryptophan, threonine, lysine, and leucine) potentially decreased lipid accumulation via increasing the fatty acid β-oxidation in maturing adipocytes, and fatty acid β-oxidation associated lipid intermediates were converted to ketone bodies by KAA. Punicalagin and KAA-loaded NLCs might have a beneficial effect in adipocytes lipolysis; KAA might stimulate the conversion of lipid intermediates into ketone bodies. This effect might be favoring the reversal of adipocyte fatty acid accumulation, hypertrophic adipocytes, which reduce the hepatic gluconeogenesis and hyperphagia in obesity associated insulin resistance conditions.

## Data Availability

The original contributions presented in the study are included in the article.

## Supplementary Materials

The following supporting information can be downloaded at: https://www.mdpi.com/article/10.3390/nano12030368/s1, Figure S1: Molecular structure of punicalagin (a), Molecular formula-C48H28O30, MW: 1084.72. Size distribution spectra (particle size) of ketogenic amino acid loaded chia seed phospholipids [NLCs-KAA] (b), punicalagin, ketogenic amino acids loaded chia seed phospholipids [NLCs-PUNI-KAA] (c) containing nano lipid carriers; Figure S2: In vitro cytotoxicity of vehicle control, PUNI and NLCs-KAA on human mesenchymal stem cells (a) and adipocytes (b) after 48 h. Each values are means ± SD (n = 6); Figure S3: Fluorescence microscopic images of propidium iodide stained vehicle control, NLC-KAA, NLC-PUNI-KAA and PUNI treated human mesenchymal stem cells after 48 h. In PI staining, the hMSCs nucleus appeared normal in all the groups; there are no signs of shrunken, pyknosis or apoptotic nucleus in NLCs-PUNI-KAA.
