# Supplementary material for: Punicalagin and Ketogenic Amino Acids Loaded Organic Lipid Carriers Enhance the Bioavailability, Mitochondrial β-Oxidation, and Ketogenesis in Maturing Adipocytes
Source: Nanomaterials (Basel). 2022 Jan 24;12(3):368. doi: 10.3390/nano12030368 (PMC8839784; doi:10.3390/nano12030368)
Supplement: Supplementary file 1 [file nanomaterials-12-00368-s001.zip › nanomaterials-1475599-supplementary.pdf]

Supplementary Materials

Punicalagin and Ketogenic Amino Acids Loaded Organic Lipid Carriers Enhance the Bioavailability, Mitochondrial β-Oxidation, and Ketogenesis in Maturing Adipocytes

Pandurangan Subash-Babu [1], Nouf Al-Numair [2,3], Tahani Almuzaini [1], Jegan Athinarayanan [1] and Ali Abdullah Alshatwi [1,*]

[1] Department of Food Science and Nutrition, College of Food and Agricultural Sciences, King Saud University, P.O. Box 2460, Riyadh 11451, Saudi Arabia; sbpandurangan@ksu.edu.sa (P.S.-B.); tooooota-nm@hotmail.com (T.A.); jathinarayanan@kse.edu.sa (J.A.)
[2] Center for Genomic Medicine, King Faisal Specialist Hospital and Research Centre, Riyadh-11451, Saudi Arabia; alnumair@kfshrc.edu.sa
[3] College of Medicine, Alfaisal University, Riyadh-11451, Saudi Arabia
* Correspondence: alshatwi@ksu.edu.sa

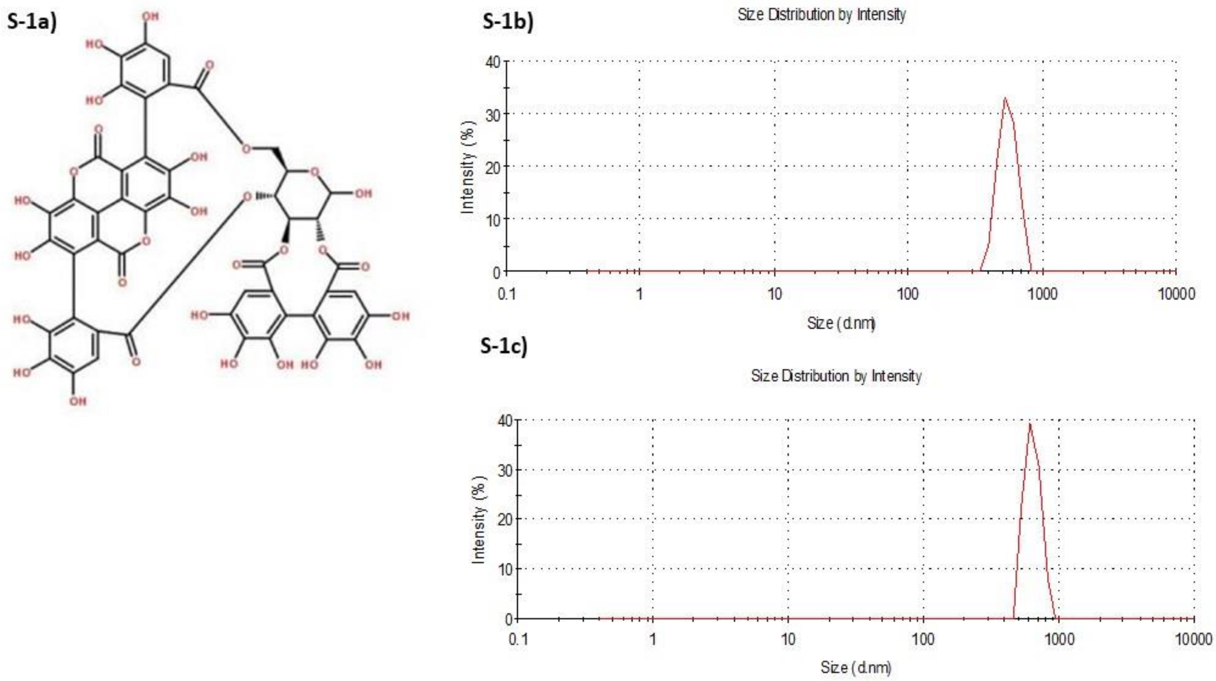

Figure S1. Molecular structure of punicalagin (**a**), Molecular formula- $C_{48}H_{28}O_{30}$, MW: 1084.72. Size distribution spectra (particle size) of ketogenic amino acid loaded chia seed phospholipids [NLCs-KAA] (**b**), punicalagin, ketogenic amino acids loaded chia seed phospholipids [NLCs-PUNI-KAA] (**c**) containing nano lipid carriers.

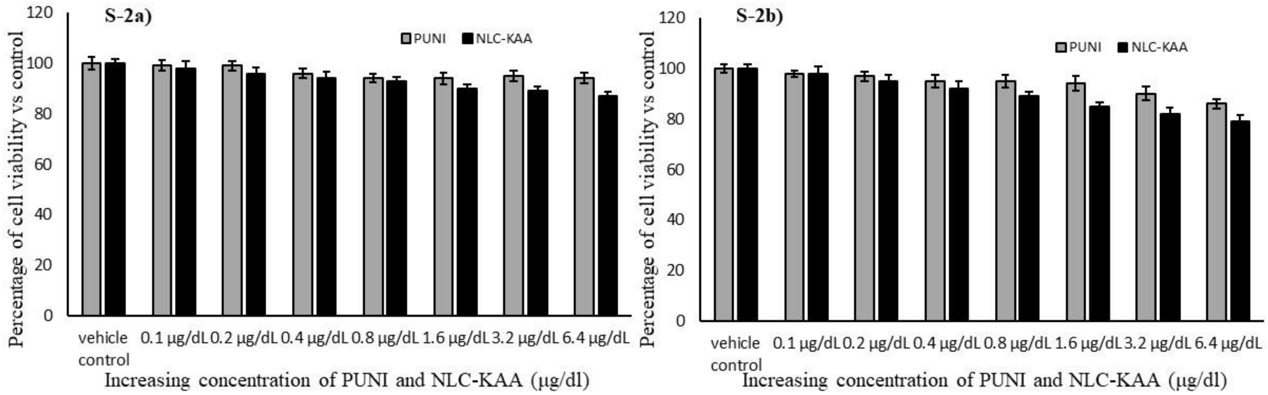

Figure S2. In vitro cytotoxicity of vehicle control, PUNI and NLCs-KAA on human mesenchymal stem cells (**a**) and adipocytes (**b**) after 48 h. Each values are means ± SD (n = 6).

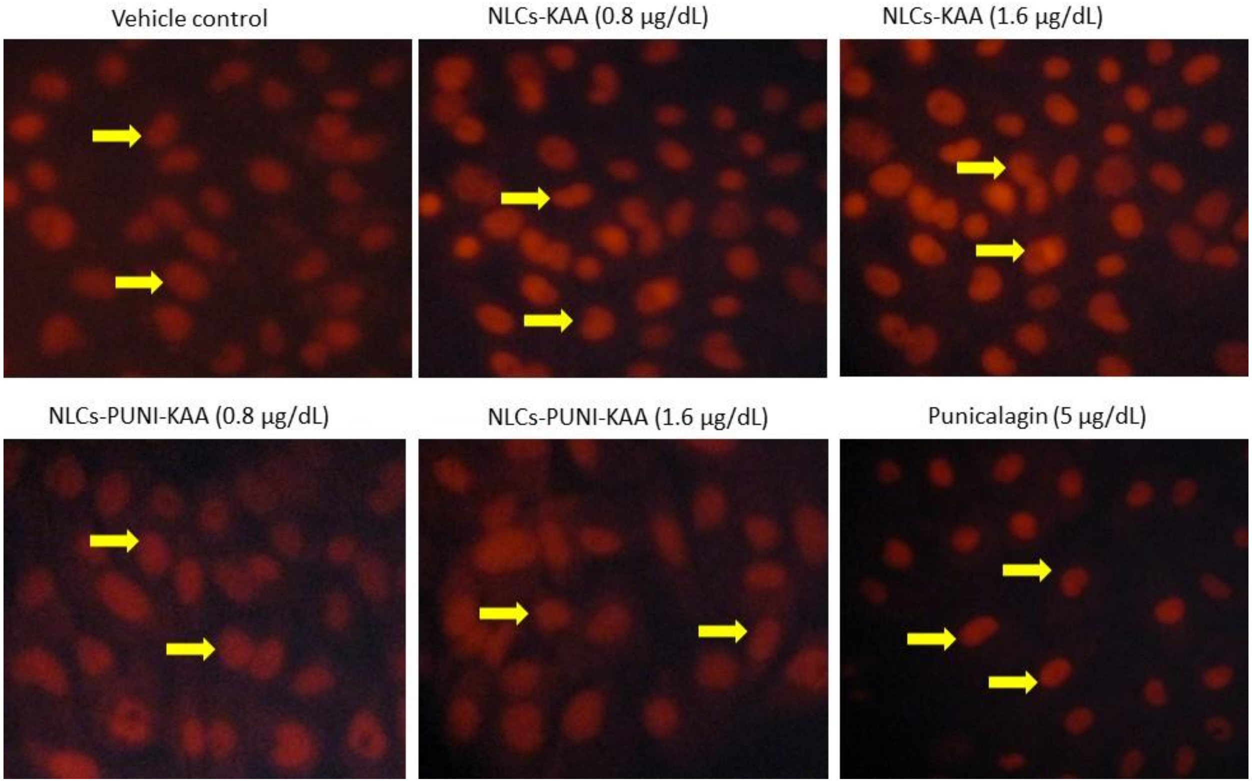

Figure S3. Fluorescence microscopic images of propidium iodide stained vehicle control, NLC-KAA, NLC-PUNI-KAA and PUNI treated human mesenchymal stem cells after 48 h. In PI staining, the hMSCs nucleus appeared normal in all the groups; there are no signs of shrunken, pyknosis, or apoptotic nucleus in NLCs-PUNI-KAA.

In propidium iodide staining, vehicle control showing normal appearance with circular shaped nucleus. NLCs-PUNI-KAA or NLCs-KAA of PUNI treated hMSCs nucleus also appeared to be normal, there are no signs of shrunken, pyknosis or apoptotic nucleus.
